# Brain Activity Changes in Cognitive Networks in Relapsing-Remitting Multiple Sclerosis – Insights from a Longitudinal fMRI Study

**DOI:** 10.1371/journal.pone.0093715

**Published:** 2014-04-09

**Authors:** Marisa Loitfelder, Franz Fazekas, Karl Koschutnig, Siegrid Fuchs, Katja Petrovic, Stefan Ropele, Alexander Pichler, Margit Jehna, Christian Langkammer, Reinhold Schmidt, Christa Neuper, Christian Enzinger

**Affiliations:** 1 Department of Neurology, Medical University of Graz, Graz, Austria; 2 Division of Neuroradiology, Department of Radiology, Medical University of Graz, Graz, Austria; 3 Institute of Psychology, Karl-Franzens-University Graz, Graz, Austria, on behalf of the Bio TechMed Initiative; University of Düsseldorf, Germany

## Abstract

**Background:**

Extrapolations from previous cross-sectional fMRI studies suggest cerebral functional changes with progression of Multiple Sclerosis (MS), but longitudinal studies are scarce. We assessed brain activation changes over time in MS patients using a cognitive fMRI paradigm and examined correlations with clinical and cognitive status and brain morphology.

**Methods:**

13 MS patients and 15 healthy controls (HC) underwent MRI including fMRI (go/no-go task), neurological and neuropsychological exams at baseline (BL) and follow-up (FU; minimum 12, median 20 months). We assessed estimates of and changes in fMRI activation, total brain and subcortical grey matter volumes, cortical thickness, and T2-lesion load. Bland-Altman (BA) plots served to assess fMRI signal variability.

**Results:**

Cognitive and disability levels remained largely stable in the patients. With the fMRI task, both at BL and FU, patients compared to HC showed increased activation in the insular cortex, precuneus, cerebellum, posterior cingulate cortex, and occipital cortex. At BL, patients vs. HC also had lower caudate nucleus, thalamus and putamen volumes. Over time, patients (but not HC) demonstrated fMRI activity increments in the left inferior parietal lobule. These correlated with worse single-digit-modality test (SDMT) performance. BA-plots attested to reproducibility of the fMRI task. In the patients, the right caudate nucleus decreased in volume which again correlated with worsening SDMT performance.

**Conclusions:**

Given preserved cognitive performance, the increased activation at BL in the patients may be viewed as largely adaptive. In contrast, the negative correlation with SDMT performance suggests increasing parietal activation over time to be maladaptive. Several areas with purported relevance for cognition showed decreased volumes at BL and right caudate nucleus volume decline correlated with decreasing SDMT performance. This highlights the dynamics of functional changes and the strategic importance of specific brain areas for cognitive processes in MS.

## Introduction

Cognitive deficits affecting memory, visuospatial skills, attention or higher executive function are frequent in multiple sclerosis (MS) and appear to elicit changes on functional MRI (fMRI) which tend to increase during the disease course [1], [2]. As such activation changes have already been noted very early in the disease and even in the absence of behaviourally detectable deficits, they have been interpreted to be at least partly adaptive to limit disease-related impairments [3], consistent with the concept of neuronal plasticity.

A previous cross-sectional study of cognitive networks in different MS-phenotypes suggested that resources for cortical reorganization might finally exhaust with progression of disease, ultimately leading to the expression of deficits [4]. Indeed, the profiles of cognitive function differed between different courses of MS in some studies [5], [6]. For the assessment of cognitive changes in MS the Symbol Digit Modalities Test (SDMT) has been reported to be most sensitive [6]–[9]. However, so far, longitudinal studies examining cognitive status with progression of MS mostly focused on morphological parameters such as lesion burden [10], [11] or brain atrophy [10], while analogue studies including fMRI are scarce [12].

We here thus sought to longitudinally assess changes in brain activity over time using repeated MRI including fMRI in non-disabled relapsing-remitting (RR) MS patients, and correlated these findings to cognitive changes (focussing on the SDMT) and MRI measurable focal tissue and regional volume changes. Specifically, we set out to explore whether and how brain function elicited by a cognitive fMRI task [4] differs in such patients from controls, and whether fMRI activation remains stable or changes dynamically over time in the patients.

## Methods

### Ethics Statement

The ethics committee on human experimentation of the Medical University of Graz approved the study. All participants gave written informed consent.

### Participants

Fifteen right-handed [13] RR-MS outpatients (**Table 1**) underwent clinical, neuropsychological, structural and functional MRI examinations at baseline (BL) and follow-up (FU) after a mandatory minimum interval of 12 months. Further inclusion criteria were clinically preserved function of the right-hand (Expanded Disability Status Scale [EDSS] [14] pyramidal score 0 or 1), sufficient visual function to recognize the stimuli and test material, and sufficient cognitive abilities to comprehend procedures. Exclusion criteria were known psychiatric disorders, clinically significant depression or fatigue, and acute relapses or steroid medication four weeks prior to the study.

**Table 1:** Characteristics of participants including clinical and morphological MRI data.

Demographics			BL vs. FU	MS vs. HC
*Sex (f/m), n*	MS	9/4	n.A.	p = 0.683^1^
	HC	9/6		
*Age (M*±*SD), years*	MS	31.3±10.0	n.A.	p = 0.110^2^
	HC	26.3±4.7		
*Education (M*±*SD), years*	MS	14.88±3.8	n.A.	p = 0.014^2^
	HC	18.17±2.7		
*Interval of examination (M*±*SD [range]), months*	MS	19.31±1.9 (14–23)	n.A.	p = 0.041^3^
	HC	20.13±0.6 (19–21)		
**Clinical parameters** (Mean, range)				
*EDSS (median [range])*	MS-BL	1.5 (0–3.5)	p = 0.336^1^	n.A.
	MS-FU	1.3 (0–3.5)		
*disease duration, years*	MS	2.55 (0.3–10.1)	n.A.	n.A.
*previous relapses, n*	MS	2.77 (1–7)	n.A.	n.A.
*annualised relapse rate during follow-up, n*	MS	0.27 (0–1.24)	n.A.	n.A.
**Structural MRI parameters** (M±SD)				
*NBV BL, cm^3^*	MS	1574.97±99.71	p = 0.171^2^	p_(BL MS vs. HC)_ = 0.060^4^
	MS	1560.38±96.71		
*NBV FU, cm^3^*	HC	1634.18±56.67	p = 0.492^2^	p_(FU MS vs. HC)_ = 0.032^4^
	HC	1629.50±63.42		
*T2-LL, cm^3^*	MS-BL	2.966±4.38	p = 0.131^2^	n.A.
	MS-FU	3.989±5.93		
*annualized BVC, [%]*	MS	−0.774±0.877	n.A.	p = 0.099^4^
	HC	−0.329±0.267		

HC = healthy controls, MS = Multiple Sclerosis, f = female, m = male, M±SD = mean±standard deviation (unless otherwise noted), EDSS = Expanded Disability Status Scale, NBV = normalized brain volume, T2-LL = T2-lesion load; BVC = brain volume change; BL = Base-Line, FU = follow up ^1^Wilcoxon-Test, ^2^paired t-Test, ^3^U-test, ^4^t-test.

Fifteen right-handed, age- and sex matched volunteers without major health problems or permanent medication with normal structural brain scans served as healthy controls (HC). As for the patients, contraindications to MRI were exclusion criteria.

### Study-related Procedures and Attrition Rates

At baseline (BL), all 30 participants (15 patients, 15 HC) underwent imaging and neuropsychological testing. At follow-up (FU), one of the 15 patients refused participation. Of the 14 remaining patients two refused neuropsychological re-testing. Another had to be excluded due to artefacts of the MRI data. Analyses of clinical and fMRI data therefore could be done for 13 patients, and concerning neuropsychological variables for 12 patients. HC also underwent repeated fMRI but were not re-examined neuropsychologically. Cognitive testing comprised the Brief Repeatable Battery of Neuropsychological Tests (BRB-N) [15] and the Wisconsin Card Sorting Test (WCST) [16]. Based on seven subtests, the BRB-N assesses information processing speed, (sustained) attention and concentration, memory, visuospatial learning, verbal learning and verbal fluency. The WCST determines higher executive abilities, such as flexibility, strategic planning and set shifting.

### MRI Data Acquisition

Imaging was performed at the same 3.0T scanner (Tim Trio, Siemens, Erlangen, Germany) using a 12-element head coil and consistent imaging protocols over the entire study period. A single-shot gradient-echo EPI-sequence (TR = 3000 ms, TE = 30 ms, FA = 90°, matrix size = 64×64, pixel size = 3.0×3.0 mm^2^) was used for fMRI, discarding the first two volumes to account for T1-saturation. Structural imaging included a high-resolution T1-weighted scan for functional image registration to precisely localize activations (3D–MPRAGE; TR = 1900 ms, TE = 2.6 ms, TI = 1900 ms; 1×1×1 mm^3^ resolution) and to calculate normalized brain volume (NBV), and a fast FLAIR sequence (TR/TE/TI = 9000 ms/69 ms/2500 ms, in plane resolution = 0.9×0.9 mm^2^, slice thickness = 3 mm) for assessment of T2-hyperintense lesion volumes.

### Functional MRI (fMRI) Paradigm

We chose an inhibition/disinhibition fMRI paradigm equivalent to own prior work [4]. During this go/no-go response-discrimination task, subjects had to react on a pre-defined target pressing a button with their right index finger. The paradigm, implemented as block design (192 volumes, duration 576 sec), consisted of ten 30 s active runs and nine interspersed 21 s rest phases (ArAr). Within active blocks, a cross and square were randomly presented in an overall approximately 1∶1 ratio. A 3-s instruction indicated the target, followed by a 3-s countdown. In every active block, one stimulus constituted the target while the other stimulus required response inhibition, ended by a 3-s “stop” signal. Targets, shown for 300 ms, had varying inter-stimulus intervals (ISI) to modulate severity (1000, 2000, 2500, 3000, 1000, 2500, 1500, 2000, 3000, and 1500 ms). Reaction times (RT), omission errors (no response although required), commission errors (false response without adequate cue), and the proportion of correct responses were recorded. Prior to scanning, participants were familiarized with the paradigm in a separate room. Broadly, the paradigm assesses integrity of response inhibition, which constitutes a higher executive function, with impact on self-evaluation and impulsivity [17].

### Analysis of Structural MRI Data

Normalized Brain Volume (NBV) and brain volume change (BVC) over time were calculated using SIENAX [18] and SIENA [19], respectively. FIRST [20] was used to obtain regional volumes of subcortical grey matter regions. All three methods are part of FMRIB’s Software Library (FSL).

FIRST [20] served to separately estimate volumes of the following subcortical regions in both hemispheres: the caudate nucleus, amygdala, hippocampus, putamen, globus pallidum, and thalamus. During registration, the input data (3D-T1 images) were transformed to the Montreal Neurological Institute (MNI) 152 standard space by affine transformations based on 12 degrees of freedom (i.e. three translations, three rotations, three scalings and three skews). After sub-cortical registration, a sub-cortical mask was applied to locate the different subcortical structures, followed by segmentation based on shape models and voxel intensities. Absolute volumes of subcortical structures were calculated, taking into account the transformations made at first stage. Finally, a boundary correction was used to determine which boundary voxels belong to the structure. All segmented subcortical regions were visually checked for errors in registration and segmentation. Further, we assessed the value of the volumes of the caudate nucleus, putamen and thalamus (as these were different between controls and patients) as predictors of SDMT performance.

FreeSurfer 5.1.0 (http://surfer.nmr.mgh.harvard.edu) served to assess cortical thickness (thk) [21]. In short, each MPRAGE image was registered to Talairach space, and subsequently grey matter (GM), white matter (WM), and pial surface were segmented. Cortical-thk was calculated as shortest distance between the boundary between WM and GM and pial surface. To realize change measurements, we used the longitudinal stream implemented in FreeSurfer [22]. An unbiased within-subject template was created for BL and FU for each participant and each measurement was registered to this template to increase reliability of thk-measurements compared with a cross-sectional design [23]. For statistical analysis, a general linear model (GLM) approach was used (Free Surfer’s QDEC, version 1.4), focusing on group differences (HC, MS) at each time point. Changes of cortical-thk (mm/year) were computed in a vertex-wise manner and smoothed using a Gaussian kernel (FWHM = 20 mm). A Monte-Carlo simulation with 500 iterations served to correct for Type 2 errors due to multiple comparisons. The significance threshold was set to p = 0.05.

T2-hyperintense lesion load (T2LL) was estimated by an experienced rater using a semiautomatic region-growing algorithm.

### Analysis of fMRI Data

Analysis was carried out using FEAT (version 5.63, www.fmrib.ox.ac.uk/fsl). Pre-statistical processing included motion correction (MCFLIRT); non-brain removal (BET); spatial smoothing (FWHM = 5 mm); and FILM for time-series statistical analysis with local autocorrelation correction. Registration to high-resolution images was carried out using FLIRT.

Higher-level analysis was done using FLAME stage 1 (FMRIB’s Local Analysis of Mixed Effects). Z (Gaussianised T/F) statistic images were thresholded using clusters determined by Z>2.3 and a (corrected) cluster significance threshold of p = 0.05 (for further details see [4]). At higher level, contrasts were calculated within-groups over time and across groups over time.

As the SDMT has been suggested as the most sensitive indicator for cognitive decline over time in MS [7], [11], we performed a correlation analysis using z-scores of this test as a regressor within the GLM. Further, to test for a possible impact of EDSS, T2LL, BVC, and length of education on brain activation at BL, respective whole-brain correlation analyses were done using demeaned parameters as a regressor within the GLM.

### General Statistics

The Statistical Package of Social Sciences (18.0.0; SPSS Inc., Chicago, IL) was used to test for inter- and intra-group differences for patients and HC, using the K-S Test for normal distribution, the U- and Wilcoxon-tests for non-parametrical comparisons, the (paired) t-Test for parametrical comparisons, and regression analysis to test for the predictive value of the SDMT on subcortical grey matter volumes.

**Figure 1:**
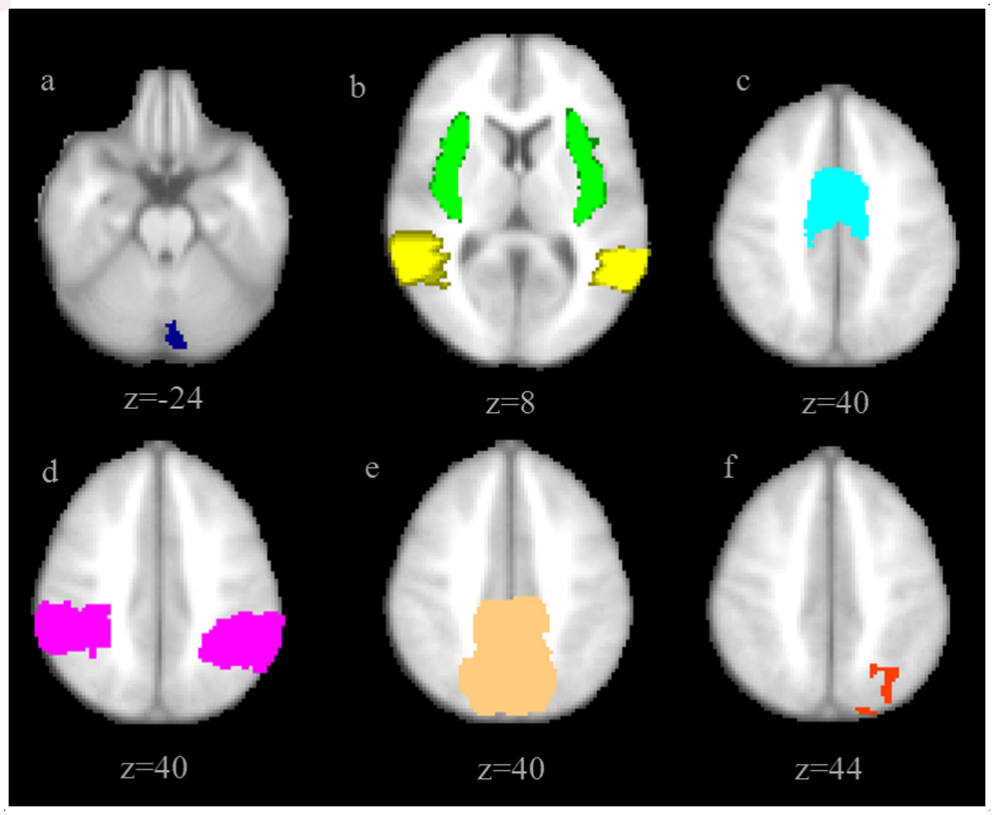
Region of interests (ROI) extracted from the Harvard-Oxford Cortical Atlas (part of FSL, b-e) and from higher level analyses (a, f) used for calculating the Bland-Altman plots. a. cerebellum crus II, b. angular gyrus (yellow), insula (green), c. supplementary motor area (SMA), d. posterior division of supramarginal gyrus, e. precuneus, f. parietal region (radiologic convention: left  =  right).

To further explore variability of the fMRI signal changes over time, to test for any systematic difference (i.e. fixed bias) between the measurements, and to identify possible outliers, Bland-Altman (BA) plots for specific region of interests (ROI) were generated for paired runs (FU-BL mean activation vs. rest) in HC and in patients. The limits of agreement were calculated using SPSS by the formula mean±1.96×standard deviation, constituting a 95% interval of agreement. BA plots measure the difference of means between runs. For repeated measurements, a mean difference of 0 indicates perfect reliability. Eight areas involved in the task (angular gyrus, insula, posterior division of supramarginal gyrus – all bilaterally - supplementary motor area (SMA), and precuneus) were extracted for ROI analyses from the Harvard-Oxford Cortical Atlas (part of FSL) and a mask for cerebellum crus II and the parietal region were hand-drawn based on functional results of the higher-level analyses (**Figure 1**). All but one ROI were exclusively used for BA plots.

## Results

### Characteristics of the Study Cohort, Clinical Profile, and Changes Over Time in the Patients

Characteristics of the study participants are presented in **Table 1** **.** Patients had experienced less years of education compared to HC. Generally, patients were in relatively early stages of the disease (**Table 1**), had low levels of disability, and had suffered 1–7 previous relapses. Overall, clinical disease activity (as expressed by relapses) over time was low, and EDSS levels remained largely stable. None of the patients converted to a secondary-progressive disease course.

### Neuropsychological Profile and Changes Over Time in the Patients

Concerning cognitive performance, at baseline, patients scored worse than HC only in a single subtest of the BRB-N (spatial recall, see **Table 2**). Otherwise, there were no significant differences between groups. Over time, at the group level, patients’ performance only significantly decreased in a single parameter of the WCST (number of correct items). Cognitive performance within the patient group was heterogeneous. According to the used definition (2 standard deviations below HCs in three or more subtests), six and four patients, respectively, were cognitively impaired at BL and at FU. Three where cognitively impaired at both time points, one demonstrated decreased, three increased, and five constant cognitive performance.

**Table 2:** Performance during neuropsychological testing (BRB-N, WCST) and the fMRI experiment (Go/no-go) for MS patients and controls at both time points.

		HC BL	HC FU	MS BL	% impaired patients BL	MS FU	% impaired patients FU	p _(HC 1 vs. MS 1)_	p _(HC 2 vs. MS 2)_	p_(MS 1 vs. MS 2)_
**BRB-N**										
	Long-term storage	64.6±4.3	n.A.	61.8±9.3	38.46	63.4±8.8	16.67	0.314^1^	n.A.	0.115^3^
	Consistent long-term retrieval	64.5±4.1	n.A.	60.2±11.1	38.46	58.9±12.4	33.33	0.209^1^	n.A.	0.889^3^
	Spatial recall test	24.2±3.6	n.A.	21.5±6.3	23.08	23.4±4.7	16.67	0.208^1^	n.A.	0.062^3^
	Symbol digit modalities test	59.7±7.9	n.A.	53.0±18.0	30.77	49.4±12.8	41.67	0.241^1^	n.A.	0.856^3^
	PASAT	50.2±7.9	n.A.	49.9±8.3	7.69	48.7±11.9	16.67	0.941^1^	n.A.	0.689^3^
	Selective reminding test (dr)	11.8±0.4	n.A.	11.4±1.0	38.46	11.3±1.3	33.33	0.470^2^	n.A.	1.000^3^
	Spatial recall test (dr)	9.3±0.8	n.A.	7.9±2.3	38.46	8.4±1.9	33.33	**0.042** ^1^	n.A.	0.221^3^
	Word list generation	26.7±5.5	n.A.	26.7±7.7	38.46	26.0±9.5	0.0	0.992^1^	n.A.	0.736^3^
**WCST**										
	Total number correct	64.7±2.9	n.A.	68.7±7.7	0.0	66.1±4.0	0.0	0.101^1^	n.A.	**0.021^3^**
	Total number of errors	8.4±2.1	n.A.	13.2±8.6	0.0	9.4±4.2	0.0	0.123^2^	n.A.	0.090^3^
	Perseverative responses	4.8±1.1	n.A.	7.2±5.0	0.0	5.5±3.0	0.0	0.168^2^	n.A.	0.256^3^
	Perseverative errors	4.8±1.1	n.A.	7.1±5.0	7.69	5.4±2.7	0.0	0.124^1^	n.A.	0.245^3^
	Nonperseverative errors	3.7±1.7	n.A.	6.2±4.5	0.0	4.1±2.4	0.0	0.082^1^	n.A.	0.054^3^
**Go/no-go**										
	Correct items, n	86.6±3.9	87.0±1.8	86.9±3.4	7.69	87.9±0.4	0.0	0.943^2^	0.265^2^	0.655^4^
	Reaction time, ms	354±71	353±75	366±54	7.69	380±61	0.0	0.661^1^	0.370^1^	0.091^3^
	Comission errors, n	2.3±2.1	1.73±2.2	1.7±3.4	7.69	2.1±2.7	0.0	0.059^2^	0.728^2^	0.891^4^
	Omisson errors, n	1.5±3.9	1.1±2.0	1.1±3.5	7.69	0.1±0.4	8.33	0.867^2^	0.265^2^	0.655^4^

HC = healthy controls, MS = Multiple Sclerosis, BL = base-line, FU = follow up, dr = delayed recall.

### Functional MRI (fMRI) Findings at Baseline and Follow-up and Changes Over Time

At both time-points, patients and HC did not differ in their behavioural performance in the scanner while confronted with the fMRI-task. There also were no significant performance decreases in both groups over time (**Table 2**). The task elicited frontotemporoparietal activation also including cerebellar regions, primarily comprising both insular cortices, the right paracingulate gyrus, the supplementary motor area (SMA), the anterior cingulate cortex (ACC), the right supramarginal gyrus, and the left precentral gyrus (cluster tables not shown, as similar to [4]).

#### Between group differences (MS vs HC)

**Figure 2:**
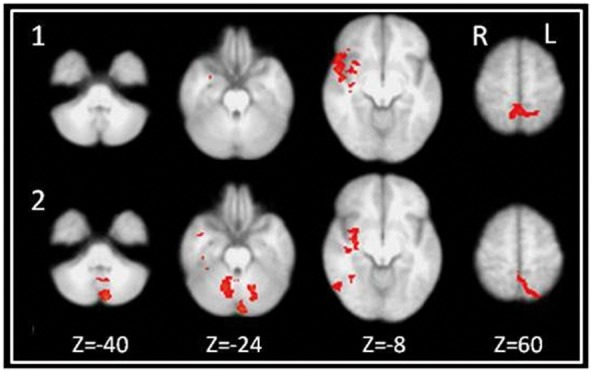
Areas with increased brain activation in MS patients vs. controls. Clusters of significant activation difference (mixed effects higher level analyses; Z>2.3; corrected cluster significance threshold p = 0.05) in contrasts for Go-/noGo task vs. rest for MS patients compared to controls at baseline (1) and follow-up (2).

Patients demonstrated increased activation compared to HC in the insular cortex and precuneus at BL. At FU, they additionally activated the posterior cingulate cortex (PCC), the cerebellum (left crus II, right lobule VI), and the lateral occipital cortex (superior and inferior division, **Figure 2** **,** **Table 3** **).** In contrast, HC showed no areas of increased activation compared to MS patients, neither at BL nor at FU.

**Table 3:** Cluster Statistics for clusters of significant differences (coordinates, maximum z-score, cluster size) between MS patients vs. healthy controls for Go−/noGo conditions vs. rest at baseline and follow-up.

	Region	L/R	x	y	z	Z-max	cluster size
*Baseline*							
	insular cortex	R	52	4	−6	3.46	1048
	precuneus	R	0	−40	52	3.39	574
*Follow-up*							
	insular cortex	R	34	−4	−12	3.69	570
	cerebellum (crus II)	L	−8	−80	−40	3.73	508
	PCC	L	−2	−36	48	3.78	483
	lateral occipital cortex, superior division	R	42	−88	10	3.52	467
	lateral occipital cortex, inferior division	R	56	−62	−6	3.36	409
	cerebellum, area VI	R	14	−60	−24	3.39	376

#### Within group differences over time

**Figure 3:**
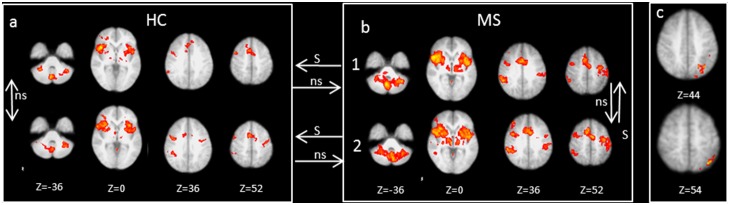
Synopsis of fMRI activations elicited by the Go-/noGo task vs. rest. Selected axial slices demonstrate brain activation in controls (HC; a) and MS patients (right; b) at baseline (upper row) and at follow-up (bottom row). The arrows indicate significant (s) and non-significant (ns) differences and their direction of contrasts between and within groups. Differences within the MS group between baseline and follow-up are displayed in c, showing increasing brain activation over time in a posterior parietal region. All images are shown in radiologic convention (mixed effects higher level analyses; Z>2.3; corrected cluster significance threshold p = 0.05).

**Figure 4:**
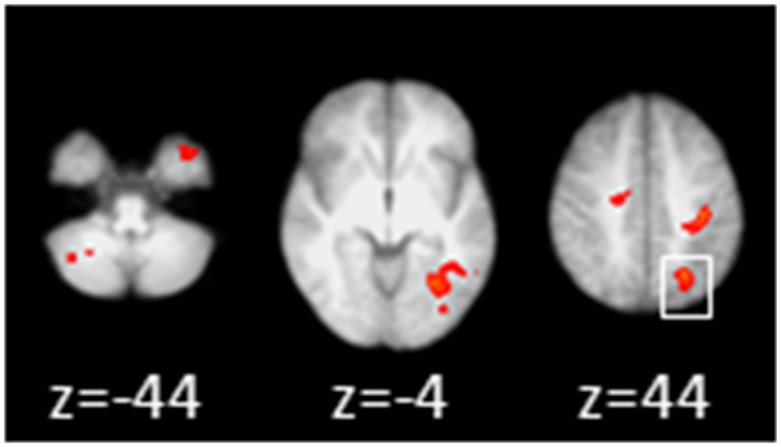
Effect of SDMT performance. Linear correlation analyses of baseline data in MS patients revealed a negative correlation in the parietal region identified to change over time in MS patients (white box), indicating that the worse SDMT performance the more activation in this region at baseline. No such an effect was identified for follow up. Additionally, negative correlation with the occipital lobe, the left precentral and the right postcentral gyrus, cerebellar regions as well as temporal areas were found.

Activation in task conditions vs. rest remained utterly stable over time in HC (**Figure 3a**). In contrast, MS patients (**Figure 3b**) demonstrated increased activation in the left inferior parietal lobule at FU (**Figure 3c**). Linear whole brain correlation analyses revealed a negative correlation between SDMT performance and activation in the left inferior parietal cortex in the patients (**Figure 4**). Worse SDMT performance also correlated with increased activation in the left lingual gyrus, left postcentral gyrus, right precentral gyrus, right cerebellum (lobule VI), and the left temporal pole.

#### Effects of EDSS, T2LL, BVC, and education duration in the patients

Linear correlation analyses of whole brain fMRI analyses in the patient group (active vs. rest conditions) with EDSS and T2LL revealed negative correlations with occipital/parietal areas, i.e. the lower the EDSS score and T2LL, the higher the activation in these areas. Some variance in functional activation in the patients also was explained by BVC - a higher change rate correlated with increasing activation in the frontal medial cortex and the SMA. At baseline, education explained variance in the functional activation only to a minor extent in the middle temporal gyrus (cluster size = 343 voxel).

#### Global/regional brain volume, focal white matter T2-lesions and changes over time

**Figure 5:**
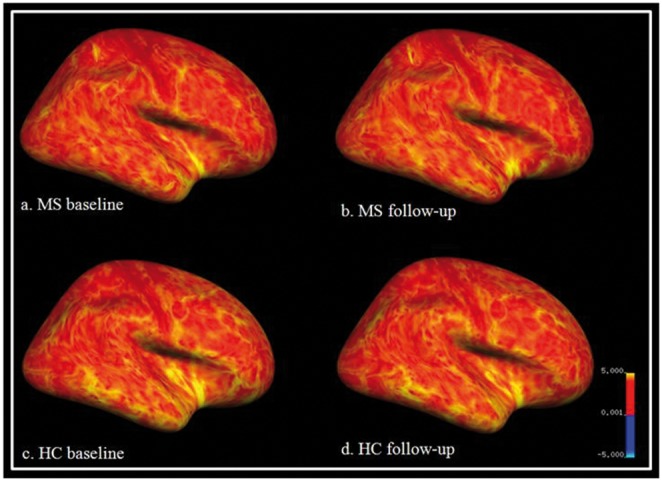
Freesurfer results showing the right inflated hemisphere in lateral view at baseline (left side; a, c) and at follow-up (right side, b, d). Upper panel: MS patients, lower panel: healthy controls (HC). Contrasts do not reveal significant differences in cortical thickness between groups. The picture illustrates average cortical thickness in mm (scale: 0.001–5.000 mm).

Between group differences (HC vs. MS). At BL, patients had a lower NBV than HC, and this difference became significant at FU (**Table 1**). Regarding subcortical regional grey matter structures, patients demonstrated lower volumes of both caudate nuclei and thalami and the right putamen (**Table 4**). In contrast, analyses of cortical thickness (**Figure 5**) did not reveal significant differences between groups. Neither the direct comparison between HC and MS at BL nor respective comparisons at FU yielded significant differences.

**Table 4:** Mean and standard deviation of subcortical grey matter regions in MS patients and healthy controls for right and left hemisphere at baseline (BL) and follow up (FU).

			HC			MS patients			
	L/R	BL/FU	mean	SD	p	mean	SD	p	p_HC vs. MS_
amygdala	L	BL	949,57	206,53	0,780	906,08	181,75	,819	,568
amygdala	L	FU	940,00	161,64		899,15	205,26		,569
amygdala	R	BL	794,57	132,98	0,449	821,08	191,16	,515	,677
amygdala	R	FU	820,50	158,09		798,00	196,97		,745
caudate	L	BL	2745,36	359,03	0,492	2368,15	472,84	,359	**,027**
caudate	L	FU	2712,29	317,40		2325,54	418,30		**,012**
caudate	R	BL	2786,79	358,90	0,326	2385,00	396,03	**,007**	**,011**
caudate	R	FU	2746,00	325,91		2276,23	355,51		**,001**
hippocampus	L	BL	2625,86	381,15	0,200	2487,46	375,73	,091	,352
hippocampus	L	FU	2674,36	389,75		2427,77	442,52		,136
hippocampus	R	BL	2617,86	386,49	0,294	2466,85	387,82	,919	,321
hippocampus	R	FU	2579,71	379,25		2470,23	342,02		,440
pallidum	L	BL	905,29	132,36	0,914	848,08	112,76	,294	,240
pallidum	L	FU	906,71	140,58		824,31	136,36		,135
pallidum	R	BL	895,86	124,59	0,929	871,92	129,89	,330	,629
pallidum	R	FU	894,21	145,80		854,31	131,40		,463
putamen	L	BL	2903,71	452,72	0,145	2622,85	414,80	,198	,106
putamen	L	FU	2823,00	508,52		2564,31	426,41		,166
putamen	R	BL	3037,93	429,45	0,085	2649,08	416,20	,052	**,025**
putamen	R	FU	2984,57	428,40		2577,85	394,41		**,017**
thalamus proper	L	BL	6011,07	839,26	0,617	5373,62	815,26	,638	,057
thalamus proper	L	FU	5985,93	739,86		5330,85	902,67		**,049**
thalamus proper	R	BL	5875,14	811,57	0,512	5137,31	760,20	,359	**,022**
thalamus proper	R	FU	5902,07	767,72		5067,85	839,41		**,012**

#### Within group differences over time

Patients did not differ significantly from HC regarding annualized global BVC over time. Similarly, comparing cortical thickness changes over time between groups, no cluster survived correction for multiple comparisons. The average volume of focal tissue abnormalities (T2-LL) in the patients was rather low and T2-LL only increased non-significantly over time. However, in the absence of significant volumetric changes of subcortical grey matter regions in HC, the volume of the right caudate nucleus decreased in MS patients from BL to FU (**Table 4**). Decreasing SDMT performance significantly predicted these volume changes (R^2^_corr_ = 0.413, p = 0.020).

### Variability of fMRI Signal Changes

**Figure 6:**
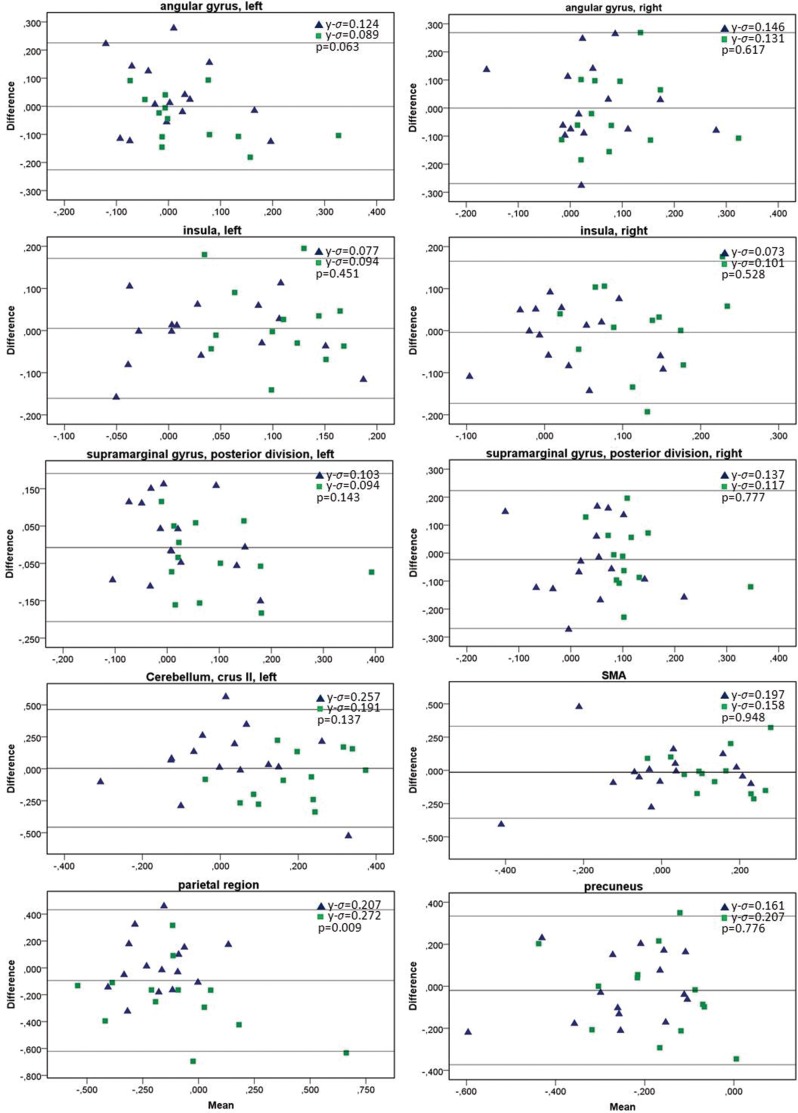
Bland-Altman plots for signal changes over time with the cognitive task in functionally relevant regions of interest. Y-sigma indicates the standard deviation on the y-axis, between group differences are calculated with the t-test. Triangles represent healthy controls, rectangles patients. For explanations, please see text.

The BA plots indicated that percent signal changes within the ten specified ROI’s (**Figure 1**) were stable (**Figure 6**), with mean differences within the limits of agreement (95%) in both the HC and MS group. For each region, three subjects at most fell outside these limits, indicating an overall high level of repeatability. Concerning all regions tested, patients did not provide more outliers than HC. Between-group comparisons of the y-standard deviation revealed only a difference for the parietal region (p = 0.009, independent t-test) in MS patients over time. All other areas yielded non-significant results. Together, these results indicate that patients did not deviate more strongly from 0 than HC (0 indicating perfect reliability).

## Discussion

We conducted a longitudinal study in non-disabled early RR-MS patients to assess functional and morphological cerebral changes over time in correlation with cognition. Results indicate extensive functional and morphological alterations already at baseline in the patients compared to controls in the absence of overtly impaired cognitive function, suggesting adaptive mechanisms. However, despite relative cognitive stability also over time, refined analyses revealed both functional and regional structural longitudinal changes correlating with SDMT performance. Together, these results highlight the dynamics of functional changes and the strategic importance of specific brain areas for cognitive processes in MS, even in a cohort with otherwise largely stable disease.

Several cross-sectional fMRI-studies [2], [24], [25] explored whether cerebral activation changes may sustain cognitive function in the context of MS pathology. Altered network allocation has been noted at the earliest stages of MS [26], with increasing deviations from normal with progression of disease [4]. However, so far, only a single longitudinal fMRI study exists on possible dynamic changes of cerebral activation patterns in MS [12], reporting on adaptive activation to improve individual working memory and processing speed. Consistently, we here also identified functional activity increments from baseline to follow-up in MS patients when compared to controls in distinct areas. Our study differs from Audoin et al.’s study [12] regarding two aspects. First, varying performance during the cognitive fMRI paradigm does not account for the fMRI activations in our study, as our paradigm successfully generated a ceiling effect. Secondly, in contrast to the cited study which used a 3 seconds version of the Paced Auditory Serial Addition Task (PASAT) for which learning effects have been reported [27], such an effect would not be expected with our approach. Against these considerations, we identified increased activation in the patients compared to controls in three widely connected cortical areas (the precuneus, the PCC, and the insular cortex).

The increased activation of the precuneus during the go/nogo paradigm confirms previous reports in RR-MS patients [4]. The precuneus and PCC are commonly considered as major components of the Default Mode Network (DMN) and often defined as “task-negative” network. Decreasing DMN activation has been noted in RR-MS [28], [29], particularly regarding the core PCC in cognitively impaired MS-patients [29]. The insular cortex has been implicated in emotion processing or regulation, but also in cognitive processes. Moreover, it has been emphasised that the right insular cortex together with the frontal cortex represent network hubs mediating between the central executive network and the DMN, and thus may be critically involved in cognitively demanding tasks [30].

Although network allocation with the fMRI task was completely stable over time in controls (supported by the BA-statistics indicating excellent repeatability of the experiment), activation increments were noted in the patients in a parietal region that negatively correlated with SDMT performance (i.e. the worse SDMT performance, the more intense the parietal activation). We therefore interpret this area as critical in SDMT processing, and its over-recruitment as maladaptive, suggesting beginning exhaustion of plasticity over time. SDMT performance has been identified as the subtest of the BRB-N most sensitive to cognitive change in MS [7].

Parallel to these functional changes, regional volume changes suggestive of deep grey matter atrophy were noted in several areas in the patients, when compared to healthy controls. At baseline, this included both caudate nuclei and thalami as well as the right putamen, in line with previous studies [9], [31]. The significant volume loss in the right caudate nucleus (but not in the thalami) in the patients represents a new finding. Together, these findings indicate that regional changes may be picked up before global brain volume changes occur and that neurodegenerative changes may have a predilection for particular areas of the brain. However, given the small patient number this assumption clearly remains speculative. However, SDMT performance decreases predicted the caudate volume at FU (but not thalamic volume; data not shown), suggesting a functional relevance of this finding. A strong association between deep grey matter volume and SDMT performance has been also been reported by others [9] which highlights this area as an interesting candidate for future research. We also examined the effect of EDSS, T2LL, BVC, and cortical thickness on functional activation. Few effects were identified, and none of these affected the main results reported.

Our study also has limitations. Although overall, the cognitive status remained stable in patients, some of them demonstrated a cognitive performance two standard deviations below the group mean. However, further subgroup analyses were precluded due to small sample size. Additional studies focusing on long term functional changes should therefore cover a wider spectrum of MS patients and stratify by cognitive dysfunction. However, the lack of existing longitudinal studies on this issue illustrates the difficulties to accomplish such investigations in clinical cohorts, and we therefore view our study as important first evidence for functional changes over time in a clinically stable subgroup of RR-MS patients. Moreover, patients and HC differed regarding education times. However, regression analysis revealed that education explained variance in activation only to a limited extent. It therefore appears unlikely that this factor accounts for main findings of our study.

Taken together, given preserved cognitive performance, we interpret the increased brain activation at BL with the cognitive task in the patients compared to controls as largely adaptive. In contrast, the negative correlation with SDMT performance suggests the parietal activation increase to be maladaptive. While the volume of several areas with purported relevance for cognition in MS was decreased at BL, only the volume decline of the right caudate nucleus correlated with decreasing SDMT performance. This suggests strategic importance of particular brain areas for cognitive processes in MS, but clearly warrants further investigations.

## References

[1] Rocca MA, Colombo B, Falini A, Ghezzi A, Martinelli V, et al (2005) Cortical adaptation in patients with MS: a cross-sectional functional MRI study of disease phenotypes. Lancet Neurol 4: 618–626. 16168930 10.1016/S1474-4422(05)70171-X

[2] Amann M, Dössegger LS, Penner I-K, Hirsch JG, Raselli C, et al (2011) Altered functional adaptation to attention and working memory tasks with increasing complexity in relapsing-remitting multiple sclerosis patients. Hum Brain Mapp 32: 1704–1719. 21077147 10.1002/hbm.21142 PMC6869921

[3] Audoin B, Au Duong M Van, Malikova I, Confort-Gouny S, Ibarrola D, et al (2006) Functional magnetic resonance imaging and cognition at the very early stage of MS. J Neurol Sci 245: 87–91. 16631203 10.1016/j.jns.2005.08.026

[4] Loitfelder M, Fazekas F, Petrovic K, Fuchs S, Ropele S, et al (2011) Reorganization in cognitive networks with progression of multiple sclerosis: insights from fMRI. Neurology 76: 526–533. 21300967 10.1212/WNL.0b013e31820b75cf

[5] Huijbregts SCJ, Kalkers NF, de Sonneville LMJ, de Groot V, Reuling IEW, et al (2004) Differences in cognitive impairment of relapsing remitting, secondary, and primary progressive MS. Neurology 63: 335–339. 15277630 10.1212/01.wnl.0000129828.03714.90

[6] Huijbregts SCJ, Kalkers NF, de Sonneville LMJ, de Groot V, Polman CH (2006) Cognitive impairment and decline in different MS subtypes. J Neurol Sci 245: 187–194. 16643951 10.1016/j.jns.2005.07.018

[7] Sonder JM, Burggraaff J, Knol DL, Polman CH, Uitdehaag BM (2013) Comparing long-term results of PASAT and SDMT scores in relation to neuropsychological testing in multiple sclerosis. Mult Scler ahead of p. 10.1177/1352458513501570 24019305

[8] Strober L, Englert J, Munschauer F, Weinstock-Guttman B, Rao S, et al (2009) Sensitivity of conventional memory tests in multiple sclerosis: comparing the Rao Brief Repeatable Neuropsychological Battery and the Minimal Assessment of Cognitive Function in MS. Mult Scler 15: 1077–1084. 19556311 10.1177/1352458509106615

[9] Batista S, Zivadinov R, Hoogs M, Bergsland N, Heininen-Brown M, et al (2012) Basal ganglia, thalamus and neocortical atrophy predicting slowed cognitive processing in multiple sclerosis. J Neurol 259: 139–146. 21720932 10.1007/s00415-011-6147-1

[10] Piras M, Magnano I, Canu E, Paulus K, Satta W, et al (2003) Longitudinal study of cognitive dysfunction in multiple sclerosis: neuropsychological, neuroradiological, and neurophysiological findings. J Neurol Neurosurg Psychiatry 74: 878–885. 12810771 10.1136/jnnp.74.7.878 PMC1738564

[11] Amato MP, Portaccio E, Goretti B, Zipoli V, Iudice A, et al (2010) Relevance of cognitive deterioration in early relapsing-remitting MS: a 3-year follow-up study. Mult Scler 16: 1474–1482. 20729256 10.1177/1352458510380089

[12] Audoin B, Reuter F, Duong MVA, Malikova I, Confort-Gouny S, et al (2008) Efficiency of cognitive control recruitment in the very early stage of multiple sclerosis: a one-year fMRI follow-up study. Mult Scler 14: 786–792. 18573836 10.1177/1352458508089360

[13] Oldfield RC (1971) The assessment and analysis of handedness: the Edinburgh inventory. Neuropsychologia 9: 97–113. 5146491 10.1016/0028-3932(71)90067-4

[14] Kurtzke JF (1983) Rating neurologic impairment in multiple sclerosis: an expanded disability status scale (EDSS). Neurology 33: 1444–1452. 6685237 10.1212/wnl.33.11.1444

[15] Boringa JB, Lazeron R HC, Reuling I EW, Ader HJ, Pfennings L, Lindeboom J, de Sonneville L, Kalkers NF PC (2001) The Brief Repeatable Battery of Neuropsychological Tests: normative values allow application in multiple sclerosis clinical practice. Mult Scler 7: 263–267. 11548987 10.1177/135245850100700409

[16] Heaton RK (1981) A manual for the Wisconsin card sorting test.

[17] Smith AM, Walker L a S, Freedman MS, DeMeulemeester C, Hogan MJ, et al (2009) fMRI investigation of disinhibition in cognitively impaired patients with multiple sclerosis. J Neurol Sci 281: 58–63. 19344919 10.1016/j.jns.2009.02.366

[18] Smith SM, Zhang Y, Jenkinson M, Chen J, Matthews PM, et al (2002) Accurate, robust, and automated longitudinal and cross-sectional brain change analysis. Neuroimage 17: 479–489. 12482100 10.1006/nimg.2002.1040

[19] Smith SM, De Stefano N, Jenkinson M, Matthews PM (n.d.) Normalized accurate measurement of longitudinal brain change. J Comput Assist Tomogr 25: 466–475. 11351200 10.1097/00004728-200105000-00022

[20] Patenaude B, Smith S, Kennedy D, Jenkinson M (2011) A Bayesian model of shape and appearance for subcortical brain segmentation. 56: 907–922. 10.1016/j.neuroimage.2011.02.046 PMC3417233 21352927

[21] Dale AM, Fischl B, Sereno MI (1999) Cortical surface-based analysis. I. Segmentation and surface reconstruction. Neuroimage 9: 179–194 doi:10.1006/nimg.1998.0395 9931268 10.1006/nimg.1998.0395

[22] Reuter M, Schmansky NJ, Rosas HD, Fischl B (2012) Within-subject template estimation for unbiased longitudinal image analysis. Neuroimage 61: 1402–1418. 22430496 10.1016/j.neuroimage.2012.02.084 PMC3389460

[23] Han X, Jovicich J, Salat D, van der Kouwe A, Quinn B, et al (2006) Reliability of MRI-derived measurements of human cerebral cortical thickness: the effects of field strength, scanner upgrade and manufacturer. Neuroimage 32: 180–194. 16651008 10.1016/j.neuroimage.2006.02.051

[24] Mainero C, Pantano P, Caramia F, Pozzilli C (2006) Brain reorganization during attention and memory tasks in multiple sclerosis: insights from functional MRI studies. J Neurol Sci 245: 93–98. 16626753 10.1016/j.jns.2005.08.024

[25] Smith AM, Walker LA, Freedman MS, DeMeulemeester C, Hogan MJ, et al (2009) fMRI investigation of disinhibition in cognitively impaired patients with multiple sclerosis. J Neurol Sci 281: 58–63. 19344919 10.1016/j.jns.2009.02.366

[26] Audoin B, Ibarrola D, Ranjeva JP, Confort-Gouny S, Malikova I, et al (2003) Compensatory cortical activation observed by fMRI during a cognitive task at the earliest stage of MS. Hum Brain Mapp 20: 51–58. 14505331 10.1002/hbm.10128 PMC6872003

[27] Cardinal KS, Wilson SM, Giesser BS, Drain AE, Sicotte NL (2008) A longitudinal fMRI study of the paced auditory serial addition task. Mult Scler 14: 465–471. 18208900 10.1177/1352458507084263

[28] Rocca M a, Valsasina P, Martinelli V, Misci P, Falini A, et al (2012) Large-scale neuronal network dysfunction in relapsing-remitting multiple sclerosis. Neurology 79: 1449–1457. 22955126 10.1212/WNL.0b013e31826d5f10

[29] Bonavita S, Gallo A, Sacco R, Corte M Della, Bisecco A, et al (2011) Distributed changes in default-mode resting-state connectivity in multiple sclerosis. Mult Scler 17: 411–422. 21239414 10.1177/1352458510394609

[30] Sridharan D, Levitin DJ, Menon V (2008) A critical role for the right fronto-insular cortex in switching between central-executive and default-mode networks. Proc Natl Acad Sci U S A 105: 12569–12574. 18723676 10.1073/pnas.0800005105 PMC2527952

[31] Lansley J, Mataix-Cols D, Grau M, Radua J, Sastre-Garriga J (2013) Localized grey matter atrophy in multiple sclerosis: a meta-analysis of voxel-based morphometry studies and associations with functional disability. Neurosci Biobehav Rev 37: 819–830. 23518268 10.1016/j.neubiorev.2013.03.006

